# Immune response analysis of solid organ transplantation recipients inoculated with inactivated COVID-19 vaccine: A retrospective analysis

**DOI:** 10.1515/med-2024-0980

**Published:** 2024-06-18

**Authors:** Jiazhi Li, Peihua Cao, Zhenhu Chen, Ruihua Deng, Yu Nie, Feixiong Pang, Xiaomian Liu, Haijia Huang, Jianrong Yang, Kebo Zhong, Yanhua Lai

**Affiliations:** Department of Transplantation, The People’s Hospital of Guangxi Zhuang Autonomous Region, Nanning 530021, Guangxi, China; Clinical Research Center, Zhujiang Hospital, Southern Medical University, Guangzhou 510280, Guangdong, China; Department of Biostatistics, School of Public Health, Southern Medical University, Guangzhou 510280, Guangdong, China; General Surgery Center, Department of Hepatobiliary Surgery II and Transformation Center for Artificial Liver, Institute of Regenerative Medicine, Zhujiang Hospital, Southern Medical University, Guangzhou 510280, Guangdong, China

**Keywords:** solid organ transplantation, COVID-19, inactivated COVID-19 vaccine, antibody, adverse events

## Abstract

**Objective:**

This study aimed to evaluate the efficacy and safety of solid organ transplantation recipients inoculated with an inactivated COVID-19 vaccine.

**Methods:**

We retrospectively analyzed the antibody levels and related adverse events of non-transplantation subjects and solid organ transplant recipients, both pre-transplantation (individuals awaiting organ transplantation) and post-transplantation (individuals who have undergone organ transplantation), who received inactivated COVID-19 vaccines from February 2021 to July 2022.

**Results:**

The study included 38 pre-transplantation vaccination group, 129 post-transplantation vaccination group, and 246 non-transplantation group. The antibody titer was assessed monthly within the period of 1–12 months after the last injection. The antibody-positive rate among the three groups were 36.84, 20.30, 61.17% (*P* < 0.05). The antibody-positive rates among three groups with one, two doses vaccine were not significantly different (*P* > 0.05), but were significantly different after three doses (*P* < 0.05). The antibody titers among three groups were significantly different after two doses (*P* < 0.05). Adverse reactions occurred in six transplant recipients, which were relieved after treatment, and not in the non-transplantation subjects.

**Conclusion:**

Inactivated COVID-19 vaccine is safe and effective for solid organ transplantation recipients, at least two doses of which should be completed before organ transplant surgery.

## 1 Introduction

Severe acute respiratory syndrome coronavirus 2 (SARS-CoV-2) is still prevalent worldwide. The coronavirus disease 2019 (COVID-19) vaccine is an important measure to prevent of SARS-CoV-2 and COVID-19 infections [1]. The cumulative number of COVID-19 vaccinations has exceeded 6 billion doses worldwide and 2.1 billion doses in China [2]. Solid organ transplantation (SOT) recipients are at high risk for COVID-19 infection, severity, and fatality, but have poor immune response of COVID-19 vaccines [3]. A number of studies have reported the application of mRNA vaccine (BNT162b2 vaccine and MRNA-1273 vaccine) and recombinant adenovirus vaccine applied in SOT recipients, but few reports have been published of SOT recipients receiving inactivated COVID-19 vaccine [4–6].

This study aimed to evaluate the immune response and safety of an inactivated COVID-19 vaccine in SOT recipients over a period of 1 year.

## 2 Materials and methods

### 2.1 Inclusion criteria and exclusion criteria

All of the inclusion criteria, but none of the exclusion criteria, were met. The following inclusion criteria were applied: age over 18 years, both sexes, and inactivated COVID-19 vaccine administration from February 2021 to July 2022 only. The exclusion criteria were as follows: HIV infection, post-transplantation <1 month, the period from vaccination to antibody detection <1 month or >12 months, and prior history of COVID-19.

The non-transplantation group included individuals who had not received organ transplants and had not the same diagnosed medical condition as the transplantation. They must meet study criteria, such as age and willingness to participate, ensuring comparability with the transplantation group.

### 2.2 Clinical data collection, research grouping, and evaluation metrics

Clinical data of patients and their families were collected by our hospital’s transplant department follow-up center through telephone or outpatient service, such as the general clinical characteristics (e.g., sex, age), comorbidity (e.g., hypertension, coronary heart disease, diabetes, systemic lupus erythematosus), transplantation type, post-transplant immune maintenance regimen, inactivated COVID-19 vaccine (e.g., vaccination time and dose, time of antibody detection), vaccine manufacturer (Sinopharm [BBIBP-CorV] or Sinovac [CoronaVac]), and vaccine-related adverse events. Vaccine-related adverse events included fever, drug allergies, muscle soreness, fatigue, dizziness, headache, nausea, rejection episodes, and graft function damage.

According to the transplant information, vaccine subjects were divided into pre-transplantation group (the SOT recipients received inactivated COVID-19 vaccine only preoperatively), post-transplantation group (SOT recipients received inactivated COVID-19 vaccine only postoperatively), and non-transplantation control group. The main evaluation metric was the antibody-positive rate, the secondary evaluation metrics were the antibody titer and adverse reaction within 1 week after vaccination.

### 2.3 Antibody detection

After each group was inoculated with 1–3 doses of the inactivated COVID-19 vaccine, the antibody titer was assessed monthly within the period of 1–12 months after the last injection. The antibody titers of SARS-CoV-2 (IgG/IgM, normal range 0–1 s/Co) were detected quantitatively by chemiluminescence (commercial kits from Zhengzhou Antu Bioengineering Co., Ltd) at our hospital. The SARS-CoV-2 antibody test was considered to be positive if the antibody titer of SARS-CoV-2 was greater than 1 s/Co, which indicated that the immune response was induced post-inoculation with an inactivated COVID-19 vaccine. The following formula was employed for the determination of the antibody-positive rate: Antibody-positive rate = Number of SARS-CoV-2 antibody-positive/total number of the group × 100%.

### 2.4 Statistical analysis

SPSS25.0 (IBM, Chicago, IL, USA) was used to analyze the data. Measurement data of normal distribution were expressed by median, and compared by two sample *t*-test. Measurement data of skewed distribution were expressed by median and quartile ranges, and compared by non-parametric tests. Categorical variable was expressed by percentages, and compared by chi-square test. Differences were considered statistically significant when *P* < 0.05.


**Ethics approval:** This work has been carried out in accordance with the Declaration of Helsinki (2000) of the World Medical Association. The study has been approved by the Ethics Committee of the People’s Hospital of Guangxi Zhuang Autonomous Region (Ethics -KY-IIT-2022-13).
**Informed consent:** All participants provided written informed consent.

## 3 Results

### 3.1 General clinical data

A total number of 378 subjects were eligible for inclusion in the study (Table 1), including 204 non-transplantation subjects with a mean age of 52 years, 151 of whom were males. The pre-transplantation group included 38 cases with a mean age of 42 years, 34 of whom were males. The post-transplantation group consisted of 129 cases with a mean age of 45 years, 94 of whom were males. In the pre-transplantation group, 14 cases received liver transplantation, and 24 cases received kidney transplantation. In the post-transplantation group, 13 patients received liver transplantation, and 116 patients received kidney transplantation. There were no significant differences in age, gender, or vaccine manufacturer among the three groups (*P* > 0.05), and the comorbidity in the non-transplantation subjects was fewer than the transplant recipients (*P* < 0.05). The time interval between the transplantation and the vaccination was from 1 month to 3 years. The immune maintenance regimen of the all SOT recipients was tacrolimus + mycophenolic acids + hormones, and the tacrolimus blood concentration was 8–10 ng/mL. The dosage of each vaccination for all subjects was 0.5 mL (Sinopharm [BBIBP-CorV] or Sinovac [CoronaVac]).

**Table 1:** General characteristics of the included participants

	Pre-transplantation group	Post-transplantation group	Non-transplantation group	*P*-value
Total number	38	129	204	—
Age (year)	42.00 (37.50, 54.50)	45.00 (39.00, 55.00)	52 (35.00, 64.25)	0.420
Male, *N* (%)	34 (89.47%)	94 (72.87%)	151 (74.02%)	0.096
Comorbidity, *N* (%)	14 (36.84%)	56 (43.41%)	15 (7.35%)	<0.05
Hypertension, *N*	13	51	14	—
Diabetes, *N*	0	7	3	—
Coronary heart disease, *N*	0	1	0	—
Systemic lupus erythematosus, *N*	1	0	0	—
Vaccine type	Inactivated COVID-19 vaccine	Inactivated COVID-19 vaccine	Inactivated COVID-19 vaccine	—
Vaccine manufacturer	—	—	—	—
First dose	—	—	—	—
Sinopharm, *N* (%)	18 (47.37%)	57 (44.19%)	110 (53.92%)	0.212
Sinovac, *N* (%)	20 (52.63%)	72 (55.81%)	94 (46.08%)	
Second dose and third dose	Sinovac	Sinovac	Sinovac	—
Transplantation type, *N* (%)	—	—	—	—
Liver transplantation	14 (36.84%)	13 (10.08%)	—	—
Kidney transplantation	24 (63.16%)	116 (89.92%)	—	—
Postoperative immune maintenance regimen	Tacrolimus + mycophenolic acids + hormones	Tacrolimus + mycophenolic acids + hormones	—	—
Systemic adverse events, *N* (%)	2 (5.26%)	4 (3.10%)	0	<0.05
Systemic adverse events, *N* (%)	2 (5.26%)	4 (3.10%)	—	0.620
Fever, *N*	2	1	0	—
Fatigue, *N*	2	1	0	—
Headache, *N*	1	0	0	—
Muscle soreness, *N*	2	1	0	—
Dizziness, *N*	0	3	0	—
Nausea and vomiting, *N*	1	1	0	—
Local adverse reaction, *N*	0	0	0	—

*N: number.

### 3.2 Antibody levels in each group with different doses within 1 year

The results of immune responses within 1 year after different doses of the inactivated COVID-19 vaccine are presented in Table 2. Both the antibody titer and antibody-positivity rate were positively correlated with the vaccination doses among the three group. There was a significant difference in the total antibody-positivity rates among the pre-transplantation group (36.84%), post-transplantation group (20.3%), and non-transplantation group (61.17%), *P* < 0.05. But there was no significant difference in the total antibody titer among the pre-transplantation group (5.08 [1.87, 6.79]), post-transplantation group (7.82 [3.83, 20.63]), and non-transplantation group (2.77 [2.3, 10.32]), *P* ＞ 0.05. The antibody-positivity rates of the pre-transplantation, post-transplantation, and the non-transplantation group with one, two, and three doses vaccine, respectively, were 0, 0, 16.67% (*P* > 0.05), 30, 8, 16.92% (*P* > 0.05), 72.73, 31.08, 84.44% (*P* < 0.05), and the median antibody titers were 0.02, 0.02, 0.015 (*P* > 0.05), 0.21, 0.04, 0.22 (*P* < 0.05), 6.32, 11.22, 4.00 (*P* < 0.05). This suggested that the antibody-positive rate of pre-transplantation group was significantly higher than the post-transplantation group after three vaccination doses, but lower than the non-transplantation group. And the antibody titer of pre-transplantation group was significantly higher than the post-transplantation group after only two vaccination doses, but lower than the non-transplantation group.

**Table 2:** Antibody levels in each group with different doses within 1 year

	Pre-transplantation group, *N* = 38	Post-transplantation group, *N* = 129	Non-transplantation group, *N* = 204	*P*-value
Antibody positive, *N* (%)	14 (36.84%)	27 (20.30%)	126 (61.17%)	<0.05
Antibody titer	5.08 (1.87, 6.79)	7.82 (3.83, 20.63)	2.77 (2.3, 10.32)	0.146
Only one dose, *N*1	7	8	6	—
Antibody positive, *N* (%)	0	0	1 (16.67%)	0.260
Antibody titer	0.02 (0.015, 0.06)	0.02 (0.013, 0.028)	0.015 (0.01, 0.035)	0.727
Only two doses, *N*2	20	49	65	—
Antibody positive, *N* (%)	6 (30.00%)	4 (8.16%)	11 (16.92%)	0.066
Antibody titer	0.21 (0.08, 1.07)	0.04 (0.01, 0.11)	0.22 (0.05, 0.75)	< 0.05
Three doses, *N*3	11	72	133	—
Antibody positive, *N* (%)	8 (72.73%)	23 (31.94%)	114 (85.71%)	<0.05
Antibody titer	6.32 (1.11, 6.93)	11.22 (2.94, 18.77)	4.00 (1.62, 9.27)	<0.05

*N: number.

### 3.3 Total antibody levels within different periods

**Figure 1:**
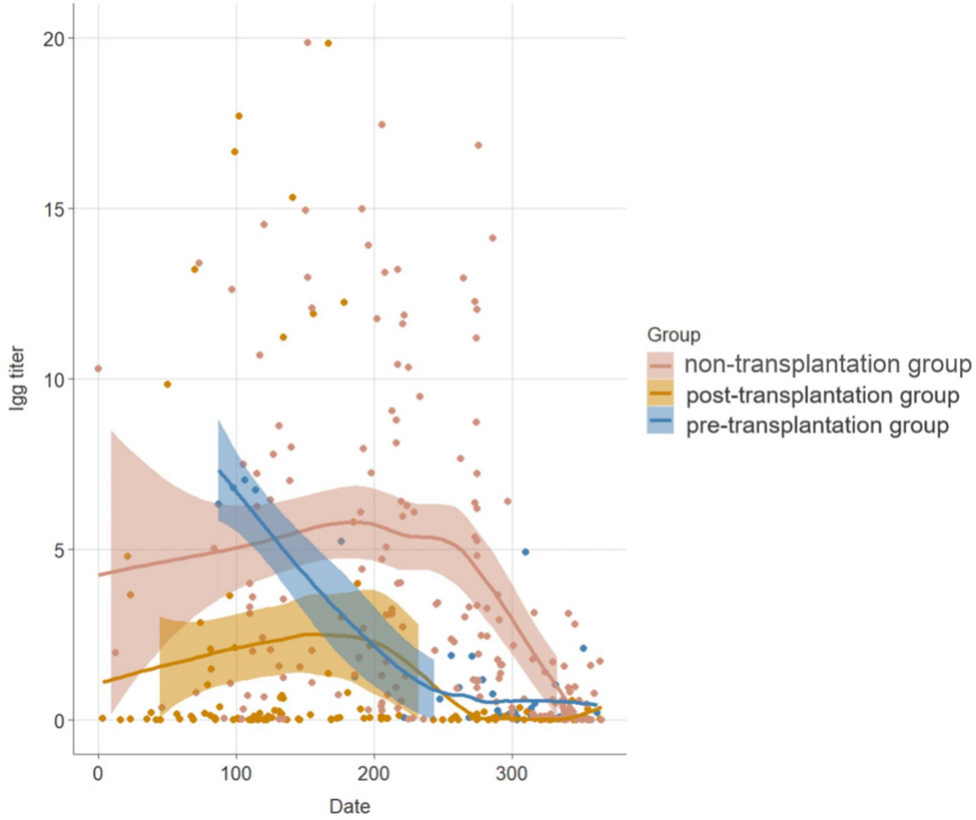
Total antibody titer curve in each group within 1 year.

The total antibody titer showed a decreasing trend in each group (including the subjects that received one, two, and three doses), but the antibody titer in the post-transplantation group decreased most significantly, reaching zero 1 year later (Figure 1). Further analysis of total antibody levels showed that there was a significant difference in the antibody-positive rate between the pre-transplantation group and post-transplantation group within different periods (*P* < 0.05) (Table 3).

**Table 3:** Total positive antibody levels within different periods

Time period	Pre-transplantation group, *N* = 14 (%)	Post-transplantation group, *N* = 27 (%)	Non-transplantation group, *N* = 126 (%)	*P*-value
From 1 to 3 months	100	35.71	100	<0.05
From 3 to 6 months	100	28.57	100	<0.05
From 6 to 9 months	28.57	16.67	80.00	<0.05
From 9 to 12 months	20.83	0	40.21	<0.05

*N: number.

### 3.4 Vaccination-related adverse events

No adverse reactions were reported in the non-transplantation group after inoculation with the inactivated COVID-19 vaccine. The rejection episodes and graft function damage were not observed in the SOT group, and there were no local adverse reactions. There were two cases of adverse reactions in the pre-transplantation group, including fever, fatigue, and headache in one case and fever, fatigue, and nausea in another. Adverse reactions occurred in four subjects in the post-transplantation group, one of whom had dizziness and muscle soreness, another complained of fever, fatigue, and nausea, and two had dizziness. The adverse reactions in the transplant recipients were more than the non-transplantation subjects (*P* < 0.05), but there were no statistically significant differences in the probability of adverse events between pre-transplantation group and post-transplantation group (*P* > 0.05). This suggested that the vaccination-related adverse events were not related to vaccination time in the transplant recipients. All adverse reactions were relieved after symptomatic treatment.

## 4 Discussion

SOT recipients are at high risk of COVID-19 infection and higher rates of severe disease and fatality after infection due to long-term immunosuppressive therapy and other comorbidities [7,8]. Earlier studies have found that the case fatality rate of COVID-19 among kidney transplantation recipients is 19–50%, which is higher than the rate of 1–5% in the general population and that of 8–15% in people aged over 70 years [9,10]. The COVID-19 case fatality rate among liver transplantation recipients was 12–19%, whereas in heart transplantation recipients it ranged from 25 to 33.3% [11–14]. A multicenter clinical study conducted in Spain established that 54.8% of 104 kidney transplantation recipients infected with COVID-19 developed acute respiratory distress syndrome (ARDS), with a case fatality rate of 27% [4]. However, previous studies found that the transplantation population was older and had higher body mass index (BMI) than healthy people, and most patients also had chronic diseases, such as hypertension, diabetes, and coronary heart disease, and all these factors could have affected the rate of severe disease and mortality of transplantation patients infected with COVID-19 [15,16]. Compared with the general population, SOT recipients have a higher rate of COVID-19 infection and post-infection severity and mortality, but whether these results are caused by immunosuppressive agents or other comorbidities needs to be further clarified by higher quality clinical studies.

The COVID-19 vaccine was still the main measure to prevent COVID-19 infection. The main types of COVID-19 vaccines received by SOT recipients include mRNA vaccine (BNT162b2 and MRNA-1273 vaccines), recombinant adenovirus vaccine, and inactivated COVID-19 vaccine. Notably, the post-vaccination antibody-positive rate in SOT recipients is generally lower than that of the general population, and the immune response to different types of vaccines is also different. A French study including 101 patients that had received liver, kidney, heart, lung, and pancreas transplantations found that the positive rate of COVID-19 antibodies in the SOT recipients was only 40% after two vaccine doses [17]. An Israeli study that enrolled 80 patients with liver transplantation established that the COVID-19 antibody-positive rate was only 47.5% after two mRNA vaccine doses [18]. Additionally, a prospective cohort study including 658 patients with liver, kidney, heart, lung, and pancreas transplantations in the United States found that the COVID-19 antibody-positive rate in SOT recipients after two vaccine doses was 54% [6]. Meanwhile, many studies have evidenced that the post-vaccination antibody-positive rate and specific T-lymphocyte count of SOT recipients increased significantly [19–21]. Other investigations revealed that the administration of an adenovirus vaccine in SOT recipients induced a lower antibody-positive rate than that of an mRNA vaccine [5]. In addition to their associations with chronic disease, immunosuppressive drugs are an important factor for the poor immune response of SOT recipients [22]. Previous studies have confirmed that the post-vaccination humoral immunity or the T-cell response of SOT recipients are significantly lower than those of the general population and dialysis patients, but the antibody-positive rate of graft recipients can be increased after administration of antimetabolic therapy [23,24].

In the present study, we found that both the antibody titer and antibody positivity were positively correlated with the vaccination doses among the three group, and the antibody-positivity rate of transplantation group was lower than the non-transplantation group. This was consistent with previous studies. Additionally, SOT recipients, inactivated at least two vaccine doses, may produce antibodies. The pre-transplantation group showed higher antibody level with decreasing drastically after 6 months, and the post-transplantation group acquired low level of antibody with sustaining the antibody in long term. The conceivable possibilities what made these phenomena of antibody level in SOT recipients were natural antibody consumption, exposure to low-dose SARS-CoV-2, and poor immune response with immunosuppression. The reason of higher titer in participants with low antibody-positive rate may be that the interval time between vaccination and antibody testing was shorter. We also found that the antibody titers among the three groups showed a decreasing trend over time, especially after 6 months. The antibody titer in the post-transplantation group reached zero 1 year later.

Moreover, we also did not find significant difference in the probability of related adverse events between pre-transplantation group and post-transplantation group inoculated with the inactivated COVID-19 vaccine, and the rejection episodes, graft function damage, related fatality in the SOT recipients. The vaccination-related adverse events in the transplant recipients were more than the non-transplantation subjects, but all were relieved after symptomatic treatment. This possible cause was immunosuppression in the SOT recipients.

In conclusion, our study suggested that the administration of an inactivated COVID-19 vaccine in SOT recipients was safe and can derive immune antibody, although this antibody-positive rate was lower than non-transplantation participants. This needed to be further investigated by large randomized clinical trials or large-scale comparative studies considering inherent selection bias and the limited number of participants. The clinic characteristics of SOT recipients inoculated with the inactivated COVID-19 vaccine, we suggest that patients with end-stage organ disease should receive at least two doses of an inactivated vaccine before undergoing organ transplantation to maintain high levels of antibodies.
